# 

**Published:** 1900-08-04

**Authors:** 


					/Duni g nr IIHII UP V/MUDUW\ T Q
cocoa A m JIEL, cocoa
ABSOLUTELY PURK. W NO DRUGS OR ALKALIES USED.
No. 723j Vol. XXVIII. Saturday,
August 4, 1900.
|~Subscnptoon^Terms,J p^ICE TWOPENCE.

				

